# Impact of Elevated Progesterone in Late Follicular Phase on Early Pregnancy Outcomes and Live Birth Rate After Fresh Embryo Transfers

**DOI:** 10.3389/fcell.2022.855455

**Published:** 2022-03-11

**Authors:** Yueming Xu, Jie Zhang, Aimin Li, Ni Yang, Na Cui, Guimin Hao, Bu-Lang Gao

**Affiliations:** Department of Reproductive Medicine, The Second Hospital of Hebei Medical University, Shijiazhuang, China

**Keywords:** elevated progesterone, *in vitro* fertilization, fresh embryo transfer, early pregnancy outcome, live birth rate

## Abstract

**Objective:** To investigate the effect of progesterone elevation during late follicular phase on early pregnancy outcomes and live births after fresh embryo transfers.

**Methods:** Patients who underwent IVF/ICSI treatment cycles were retrospectively enrolled. The effect of progesterone elevation was analyzed on early pregnancy outcome and live births after fresh embryo transfers.

**Results:** A total of 2,404 patients were enrolled on the day of HCG triggering (HCG0), 1,584 patients on the day before HCG triggering (HCG-1), and 800 patients 2 days before HCG triggering (HCG-2). With a 1 ng/ml increase in the progesterone level on HCG0 day when the progesterone level was ≥1.5 ng/ml, the clinical pregnancy rate decreased by 60% (95% CI: 0.2–0.7, *p* = 0.004), the intrauterine pregnancy rate decreased by 70% (95% CI: 0.2–0.7, *p* = 0.003), and the live birth rate decreased by 70% (95% CI: 0.1–0.7, *p* = 0.004). With a 1 ng/ml increase in the progesterone level on HCG-1 day, the clinical pregnancy rate decreased by 90% (95% CI: 0.0–0.5, *p* = 0.003) when the progesterone level was ≥1.6 ng/ml, the intrauterine pregnancy rate decreased by 90% (95% CI: 0.0–0.5, *p* = 0.001) when the progesterone was ≥1.5 ng/ml, and the live birth rate decreased by 90% (95% CI: 0.0–0.6, *p* = 0.015) when the progesterone was ≥1.7 ng/ml. On HCG-2 day when the progesterone was ≥1.2 ng/ml, the clinical pregnancy rate decreased by 80% (95% CI: 0.1–0.6, *p* = 0.003), and the intrauterine pregnancy rate decreased by 70% (95% CI: 0.1–0.7, *p* = 0.007) with a 1 ng/ml increase in the progesterone level.

**Conclusion:** Elevated progesterone level during the late follicular phase is an independent risk factor affecting the clinical pregnancy rate, intrauterine pregnancy rate, and live birth rate among infertile patients undergoing IVF/ICSI after fresh embryo transfers. When the progesterone level exceeds a certain level, the early pregnancy and live birth rates after fresh embryo transfers show a rapid downward trend.

## Introduction

For more than 2 decades, the effect of elevated progesterone on the outcome of *in vitro* fertilization and embryo transfer (IVF-ET) has been the focus of intensive scientific research. Although the application of gonadotropin-releasing hormone (GnRH) analogues could significantly reduce the incidence of premature luteinization in controlled ovarian stimulation (COS), the incidence of progesterone elevation in late follicular phase still fluctuated at 5%–30%. Since Edelstein et al. published their first report in 1990 (Edelstein et al., 1990), there have been many studies investigating the effect of elevated progesterone during the late follicular phase on IVF-ET outcomes with conflicting conclusions (Arvis et al., 2019).

To our knowledge, the most commonly used cutoff value for elevated progesterone on the day of human chorionic gonadotrophin (HCG) administration is 1.5 ng/ml (Bosch et al., 2010; Venetis et al., 2013). However, this cutoff value of progesterone elevation should be treated with caution because of the high variability of assays used for progesterone measurement, making it difficult to compare the results of different studies (Bosch et al., 2003; Kiliçdag et al., 2010; Santos-Ribeiro et al., 2014; Connell et al., 2016; Cui et al., 2017). In most of these studies, the serum progesterone concentration had been divided into different levels, and in the current study, the appropriate serum progesterone threshold that negatively affected the IVF outcome was investigated without dividing the serum concentration of progesterone into different levels, given that the progesterone level is a continuous variable. The second objective of this study was to evaluate the effect of elevated progesterone levels on the pregnancy outcomes of IVF-ET in the late follicular phase and the live birth rate.

## Materials and Methods

### Subjects

This was a retrospective, one-center case-cohort study on patients undergoing routine IVF-ET. The study protocol was approved by the Medical Ethics Committee of the Second Hospital of Hebei Medical University (No. 2017-P033). The criteria for inclusion were as follows: (1) patients undergoing IVF/intracytoplasmic sperm injection (ICSI) for the first time, (2) age ≤38 years, and (3) fresh embryo transfers. The criteria for exclusion were as follows: (1) abnormal uterine development, (2) preimplantation genetic testing (PGT), (3) endocrine diseases, and (4) missing progesterone values and unknown live birth at follow-up. A total of 2,404 patients undergoing IVF/ICSI treatment at our hospital from January 2018 to December 2019 were enrolled.

### Controlled Ovarian Stimulation Protocol

The patients underwent COS with the use of luteal phase short-acting long protocol, GnRH antagonist protocol, and long-acting long protocol at the early follicular stage. According to the patient’s ovarian reserve and body mass index (BMI), ovarian stimulation was started with administration of 150–300 IU/day recombinant FSH (Gonal-F; Serono, Pharma S. p.A., Bari, Italy). The development of follicles was monitored by transvaginal ultrasonography. HCG 6500–10000U was given to trigger ovulation when two leading follicles reached a mean diameter of 18 mm. Oocytes were retrieved transvaginally 36–38 h after HCG administration. IVF or ICSI was performed according to the cause of infertility in the patients. Fewer than two embryos were transferred on day 3 after oocyte retrieval if the serum progesterone level was ≤2.0 ng/ml on the day of HCG administration, and excessive good-quality embryos were cryopreserved. Patients received conventional corpus luteum support, and embryo transfer was performed 3 days after oocyte retrieval. Preliminary diagnosis of biochemical pregnancy was made based on blood *β*-HCG 14 days after transplantation. Clinical pregnancy indicated the gestational sac, fetal bud, and fetal heart beat on ultrasound imaging approximately 30 days after transplantation.

### Hormone Measurements

Whole blood was collected between 7:30 and 9 a.m. Serum follicle stimulating hormone (FSH), luteinizing hormone (LH), estradiol (E2), and progesterone were measured using the Immulite 2000 Immunoassay System (Siemens Healthcare Global, Munich, Germany) throughout the research process to minimize the bias in results related to time and reagent batch updates.

### Indicators

The laboratory indicators included number of oocytes, number of two pronuclear (2 PN) embryos, number of normal cleavage embryos (2 PN cleavage), number of embryos transferred, and number of available embryos (the sum of number of transferred embryos and frozen embryos).

The clinical indicators included age, BMI, anti-Müllerian hormone (AMH), basic FSH level (bFSH), number of antral follicles (AFC), infertility factors (tubal factor infertility, polycystic ovary syndrome, endometriosis, primary ovarian insufficiency, ovulation disorder, male factor, and unknown factors), diagnosis (primary infertility and secondary infertility), COS protocols (luteal phase short-acting long protocol, GnRH antagonist protocol, and long-acting long protocol at early follicular stage), average Gn dosage, clinical pregnancy rate (clinical pregnancy cycles/transplantation cycles × 100%), intrauterine pregnancy rate (intrauterine pregnancy cycles/transplantation cycles × 100%), abortion (termination of pregnancy for less than 28 weeks of pregnancy and less than 1,000 g of fetus weight) rate (abortion cycles/transplantation cycles × 100%), early abortion (abortion occurring before 12 weeks of pregnancy) rate (early abortion cycles/transplantation cycles × 100%), and live birth rate (live birth cycles/transplantation cycles × 100%).

### Statistical Analysis

All statistical analyses were performed with the Empower (R) (X&Y solutions, Boston, MA, United States) and the SPSS 23.0 statistical software (IBM, Chicago, IL, United States ) (Yu et al., 2014; Park et al., 2017; Sun et al., 2020). Continuous variables of normal distribution were expressed as the mean ± standard deviation (mean ± SD), and variables of non-normal distribution were presented as median ± quartile range (median ± QR). Classified variables were expressed as frequency and percentage (%). The independent sample *t*-test was used to analyze the continuous variables, whereas the odds ratio (OR) and chi-square test were used to analyze the categorical variables. The covariate screening, regression analysis, and curve fitting were performed using the Empower (R) statistical software. Univariate regression analysis was performed to select confounding factors, and multivariate logistic regression analysis was performed to adjust confounding factors for the effect of elevated progesterone on pregnancy outcomes. A generalized additive model was then applied to estimate the independent relationship between the progesterone level and pregnancy outcomes, with adjustment for potential confounders. A two-piece-wise linear regression model was further applied to examine the threshold effect of progesterone level on pregnancy outcomes according to the smoothing plot. The turning point of progesterone level where the relationship between the pregnancy outcomes and progesterone level started to change significantly was determined using the trial method, which was to move the trial turning point along the pre-defined interval and pick up the one that gave the maximal model likelihood. The cutoff value was determined in combination with the threshold effect analysis. *p* < 0.05 was considered statistically significant.

## Results

### Univariate Analysis of Pregnancy Outcomes

Based on the inclusion criteria, 2,404 patients were enrolled on the day of HCG triggering (HCG0), 1,584 patients on the day before HCG triggering (HCG-1), and 800 patients 2 days before HCG triggering (HCG-2). No significant (*p* > 0.05) differences were detected in the baseline data among three groups (Table 1).

**TABLE 1:** Demography and information of different groups.

	Statistics	HCG-1 (*n* = 1,584)	HCG-2 (*n* = 800)
	HCG0 (*n* = 2,404)		
Age (years)	30.1 ± 3.8	30.2 ± 3.8	30.2 ± 4.0
BMI (kg/m^2^)	23.6 ± 3.7	23.8 ± 3.8	23.8 ± 3.8
AMH (ng/ml)	3.4 ± 2.5	3.5 ± 2.6	3.4 ± 2.6
bFSH (mIU/ml)	7.6 ± 2.8	7.7 ± 2.9	7.7 ± 2.5
AFC	12.6 ± 5.9	12.6 ± 6.0	12.4 ± 5.8
Infertility factors (%)			
Tube	57.4	56.2	58.5
PCOS	4.4	5	4.6
Endometriosis	3.1	3.3	2.8
POI	2.5	2.7	2.1
Ovulation disorder	1.2	1.5	0.8
Male	29.9	30	29.4
Unknown	1.5	1.5	1.9
Diagnosis (%)			
Primary infertility	52.7	54	51.1
Secondary infertility	47.3	46	48.9
Protocol (%)			
GnRH-a	42.9	39.1	39.5
GnRH-ant	18.9	20.6	20.8
Long GnRH-a	38.2	40.3	39.7
Average Gn dose(IU/day)	206.8 ± 50.1	207.8 ± 50.2	210.0 ± 48.9
No. of oocytes	10.8 ± 5.0	10.8 ± 4.9	10.7 ± 4.9
No. of 2 PN	6.7 ± 3.8	6.7 ± 3.8	6.6 ± 3.7
No. of 2 PN cleavage	6.6 ± 3.7	6.6 ± 3.7	6.5 ± 3.6
No. of available embryos	3.2 ± 1.9	3.2 ± 1.9	3.2 ± 1.8
No. of ET	1.9 ± 0.3	1.9 ± 0.3	1.9 ± 0.2
Progesterone (ng/ml)	1.1 ± 0.5	1.0 ± 0.4	0.9 ± 0.4

AMH, anti-Müllerian hormone; AFC, antral follicles; bFSH, basic follicle stimulating hormone; BMI, body mass index; GnRH-a, gonadotropin-releasing hormone analogue; GnRH-ant, GnRH, antagonist; PCOS, polycystic ovary syndrome; POI, primary ovarian insufficiency; 2 PN, two pronuclear embryos; ET, embryo transfer. *p < 0.05.

To determine the risk factors for pregnancy outcomes, a logistic regression model was established and analyzed. Clinical pregnancy rate, intrauterine pregnancy rate, early abortion rate, and live birth rate were used as the dependent variable, respectively. Different risk factors identified in the univariable analysis were used as independent variables (Tables 2–6).

**TABLE 2:** Risk factors for clinical pregnancy.

Risk factors	Clinical pregnancy rate	OR (95% CI), *p*	
	HCG0 (*n* = 2,404)	HCG-1 (*n* = 1,584)	HCG-2 (*n* = 800)
Age (years)	1.0 (1.0, 1.0), 0.055	1.0 (0.9, 1.0), 0.052	1.0 (0.9, 1.0), 0.054
BMI (kg/m^2^)	1.0 (1.0, 1.0), 0.457	1.0 (1.0, 1.0), 0.600	1.0 (1.0, 1.1), 0.498
AMH (ng/ml)	1.0 (1.0, 1.1), 0.295	1.0 (1.0, 1.1), 0.364	1.0 (0.9, 1.0), 0.523
bFSH (mIU/ml)	1.0 (1.0, 1.0), 0.292	1.0 (0.9, 1.0), 0.318	1.0 (1.0, 1.1), 0.271
AFC	1.0 (1.0, 1.0)*, <0.001	1.0 (1.0,1.0)*, 0.002	1.0 (1.0, 1.1)*, 0.017
Infertility factors (%)			
Tube	1.0	1.0	1.0
PCOS	1.4 (0.9, 2.1), 0.116	1.6 (1.0, 2.6)*, 0.045	1.8 (0.9, 3.6), 0.093
Endometriosis	1.3 (0.8, 2.1), 0.282	1.0 (0.6, 1.8), 0.875	1.3 (0.6, 3.1), 0.528
POI	1.1 (0.7, 1.9), 0.658	1.0 (0.6, 1.9), 0.887	1.2 (0.5, 3.3), 0.668
Ovulation disorder	1.3 (0.6, 2.6), 0.553	1.6 (0.7, 3.8), 0.260	0.5 (0.1, 3.0), 0.492
Male	1.3 (1.1, 1.6)*, 0.002	1.4 (1.1, 1.8)*, 0.003	1.6 (0.6, 4.7), 0.351
Unknown	1.2 (0.6, 2.3), 0.593	1.1 (0.5, 2.6), 0.755	1.4 (1.0, 1.9)*, 0.033
Diagnosis(%)			
Primary infertility	1.0	1.0	1.0
Secondary infertility	0.9 (0.8, 1.0), 0.144	0.9 (0.7, 1.1), 0.187	0.8 (0.6, 1.0), 0.080
Protocol(%)			
GnRH-a	1.0	1.0	1.0
GnRH-ant	0.9 (0.7, 1.1), 0.212	0.9 (0.7, 1.1), 0.342	0.9 (0.6, 1.4), 0.751
Long GnRH-a	1.0 (0.9, 1.2), 0.853	1.1 (0.9, 1.3), 0.501	1.1 (0.8, 1.5), 0.429
Average Gn dose (IU/day)	1.0 (1.0, 1.0), 0.062	1.0 (1.0, 1.0)*, 0.035	1.0 (1.0, 1.0), 0.656
No. of oocytes	1.0 (1.0, 1.0)*, <0.001	1.0 (1.0, 1.1)*, 0.002	1.0 (1.0, 1.0), 0.516
No. of 2 PN	1.0 (1.0, 1.1)*, <0.001	1.0 (1.0, 1.1)*, 0.003	1.0 (1.0, 1.1), 0.177
No. of 2 PN cleavage	1.0 (1.0, 1.1)*, <0.001	1.0 (1.0, 1.1)*, 0.003	1.0 (1.0, 1.1), 0.191
No. of available embryos	1.1 (1.1, 1.2)*, <0.001	1.1 (1.1, 1.2)*, <0.001	1.2 (1.1, 1.2)*, <0.001
No. of ET	2.4 (1.7, 3.4)*, <0.001	2.6 (1.7, 3.8)*, <0.001	2.9 (1.5, 5.4)*, 0.001
Progesterone (ng/ml)	0.9 (0.7, 1.0), 0.069	0.8 (0.6, 1.0)*, 0.049	0.7 (0.5, 1.0)*, 0.024

AMH, anti-Müllerian hormone; AFC, antral follicles; bFSH, basic follicle stimulating hormone; BMI, body mass index; CI, confidence interval; GnRH-a, gonadotropin-releasing hormone analogue; GnRH-ant, GnRH, antagonist; OR, odds ratio; PCOS, polycystic ovary syndrome; POI, primary ovarian insufficiency; 2 PN, two pronuclear embryos; ET, embryo transfer. *p < 0.05.

**TABLE 3:** Risk factors for intrauterine pregnancy rate.

	Intrauterine pregnancy rate	OR (95% CI), *p*	
Risk factors	HCG0 (*n* = 2,404)	HCG-1 (*n* = 1,584)	HCG-2 (*n* = 800)
Age (years)	−0.0 (-0.0,-0.0)*, 0.045	−0.0(-0.0,-0.0)*, 0.035	−0.0(-0.0,-0.0)*, 0.028
BMI (kg/m^2^)	0.0 (−0.0, 0.0), 0.722	0.0 (−0.0, 0.0), 0.908	0.0 (−0.0, 0.0), 0.851
AMH (ng/ml)	0.0 (−0.0, 0.0), 0.206	0.0 (−0.0, 0.0), 0.282	−0.0 (−0.0, 0.0), 0.647
bFSH (mIU/ml)	−0.0 (−0.0,0.0), 0.200	−0.0 (−0.0,0.0), 0.224	0.0 (−0.0, 0.0), 0.323
AFC	0.0 (0.0, 0.0)*, <0.001	0.0 (0.0, 0.0)*, <0.001	0.0 (0.0, 0.0)*, 0.016
Infertility factors (%)			
Tube	0	0	0
PCOS	0.1 (−0.0, 0.2), 0.074	0.1 (0.0, 0.2)*, 0.028	0.2 (−0.0, 0.3), 0.064
Endometriosis	0.1 (−0.1, 0.2), 0.300	0.0 (−0.1, 0.1), 0.976	0.1 (−0.1, 0.3), 0.453
POI	0.0 (−0.1, 0.2), 0.545	0.0 (−0.1,0.2), 0.786	0.1 (−0.2, 0.3), 0.594
Ovulation disorder	0.1 (−0.1, 0.3), 0.479	0.1 (−0.1, 0.3), 0.217	−0.1 (−0.5, 0.3), 0.525
Male	0.1 (0.0, 0.1)*, 0.002	0.1 (0.0, 0.1)*, 0.004	0.1 (−0.1, 0.4)*, 0.025
Unknown	0.1 (−0.1, 0.2), 0.507	0.0 (−0.2, 0.2), 0.683	0.1 (0.0, 0.2), 0.298
Diagnosis (%)			
Primary infertility	0	0	0
Secondary infertility	−0.0 (−0.1,0.0), 0.141	−0.0 (−0.1,0.0), 0.138	−0.1 (−0.1, 0.0), 0.057
Protocol (%)			
GnRH-a	0	0	0
GnRH-ant	−0.0 (−0.1,0.0), 0.093	−0.1 (−0.1,0.0), 0.128	−0.0 (−0.1, 0.1), 0.620
Long GnRH-a	0.0 (−0.0, 0.0), 0.639	0.0 (−0.0, 0.1), 0.397	0.0 (−0.0, 0.1), 0.384
Average Gn dose (IU/day)	−0.0 (−0.0,−0.0)*, 0.026	−0.0(−0.0,−0.0)*, 0.017	−0.0 (−0.0, 0.0), 0.527
No. of oocytes	0.0 (0.0, 0.0)*, <0.001	0.0 (0.0, 0.0)*, 0.001	0.0 (−0.0, 0.0), 0.406
No. of 2 PN	0.0 (0.0, 0.0)*, <0.001	0.0 (0.0, 0.0)*, 0.004	0.0 (−0.0, 0.0), 0.172
No. of 2 PN cleavage	0.0 (0.0, 0.0)*, <0.001	0.0 (0.0, 0.0)*, 0.004	0.0 (−0.0, 0.0), 0.183
No. of available embryos	0.0 (0.0, 0.0)*, <0.001	0.0 (0.0, 0.0)*, <0.001	0.0 (0.0, 0.1)*, 0.001
No. of ET	0.2 (0.1, 0.3)*, <0.001	0.2 (0.1, 0.3)*, <0.001	0.2 (0.1, 0.4)*, 0.001
Progesterone (ng/ml)	0.8 (0.7, 1.0)*, 0.037	−0.0 (−0.1,−0.0)*, 0.018	−0.1 (−0.2, −0.0)*, 0.025

AMH, anti-Müllerian hormone; AFC, antral follicles; bFSH, basic follicle stimulating hormone; BMI, body mass index; CI, confidence interval; GnRH-a, gonadotropin-releasing hormone analogue; GnRH-ant, GnRH, antagonist; Inf, Infinity; OR, odds ratio; PCOS, polycystic ovary syndrome; POI, primary ovarian insufficiency; 2 PN, two pronuclear embryos; ET, embryo transfer. *p < 0.05.

**TABLE 4:** Risk factors for live birth rate.

Risk factors	Live birth rate	OR (95% CI), *p*	
	HCG0 (*n* = 2,404)	HCG-1 (*n* = 1,584)	HCG-2 (*n* = 800)
Age (years)	1.0 (0.9, 1.0)*, <0.001	1.0 (0.9, 1.0)*, <0.001	1.0 (0.9, 1.0)*, 0.023
BMI (kg/m^2^)	1.0 (1.0, 1.0), 0.545	1.0 (1.0, 1.0), 0.497	1.0 (0.9, 1.0), 0.436
AMH (ng/ml)	1.0 (1.0, 1.1)*, 0.037	1.0 (1.0, 1.1)*, 0.032	1.0 (1.0, 1.1), 0.622
bFSH (mIU/ml)	1.0 (0.9, 1.0), 0.210	1.0 (0.9, 1.0),0.073	1.0 (0.9, 1.1), 0.868
AFC	1.0 (1.0, 1.0)*, 0.003	1.0 (1.0, 1.0)*, <0.001	1.0 (1.0, 1.1)*, 0.028
Infertility factors (%)			
Tube	1.0	1.0	1.0
PCOS	1.1 (0.8, 1.7), 0.530	1.4 (0.8, 2.2),0.208	1.2 (0.6, 2.4), 0.620
Endometriosis	1.2 (0.7, 1.9), 0.529	1.1 (0.6, 1.9), 0.867	1.3 (0.5, 3.1), 0.611
POI	1.0 (0.6, 1.7), 0.987	0.9 (0.5, 1.7), 0.734	0.7 (0.2, 2.1), 0.504
Ovulation disorder	1.4 (0.7, 2.9), 0.395	1.8 (0.8, 4.2), 0.157	0.4 (0.1, 3.8), 0.457
Male	1.2 (1.0, 1.5)*, 0.019	1.3 (1.0, 1.7)*, 0.021	2.5 (0.9, 7.1)*, 0.036
Unknown	1.3 (0.7, 2.6), 0.394	1.1 (0.4, 2.5), 0.896	1.4 (1.0, 2.0), 0.079
Diagnosis (%)			
Primary infertility	1.0	1.0	1.0
Secondary infertility	0.9 (0.8, 1.1), 0.529	0.9 (0.7, 1.1), 0.212	1.0 (0.7, 1.3), 0.993
Protocol (%)			
GnRH-a	1.0	1.0	1.0
GnRH-ant	0.7 (0.5, 0.8)*, <0.001	0.7 (0.5, 0.9)*, 0.011	0.7 (0.5, 1.1), 0.085
Long GnRH-a	1.4 (1.2, 1.7)*, <0.001	1.5 (1.2, 1.9)*, <0.001	1.3 (1.0, 1.9), 0.079
Average Gn dose (IU/day)	1.0 (1.0, 1.0)*, <0.001	1.0 (1.0, 1.0)*, <0.001	1.0 (1.0, 1.0), 0.054
No. of oocytes	1.0 (1.0, 1.1)*, <0.001	1.0 (1.0, 1.1)*, <0.001	1.0 (1.0, 1.1), 0.141
No. of 2 PN	1.0 (1.0, 1.1)*, <0.001	1.0 (1.0, 1.1)*, 0.002	1.0 (1.0, 1.1), 0.100
No. of 2 PN cleavage	1.0 (1.0, 1.1)*, <0.001	1.0 (1.0, 1.1)*, 0.002	1.0 (1.0, 1.1), 0.135
No. of available embryos	1.1 (1.1, 1.1)*, <0.001	1.1 (1.0, 1.2)*, <0.001	1.1 (1.0, 1.2), 0.071
No. of ET	2.7 (1.8, 4.1)*, <0.001	2.6 (1.6, 4.2)*, <0.001	2.1 (1.1, 4.4)*, 0.035

AMH, anti-Müllerian hormone; AFC, antral follicles; bFSH, basic follicle stimulating hormone; BMI, body mass index; CI, confidence interval; GnRH-a, gonadotropin-releasing hormone analogue; GnRH-ant, GnRH, antagonist; OR, odds ratio; PCOS, polycystic ovary syndrome; POI, primary ovarian insufficiency; 2 PN, two pronuclear embryos; ET, embryo transfer. *p < 0.05.

**TABLE 5:** Risk factors for abortion rate.

Risk factors	Abortion rate	OR (95% CI), *p*	
	HCG0 (*n* = 2,404)	HCG-1 (*n* = 1,584)	HCG-2 (*n* = 800)
Age (years)	1.0 (1.0, 1.1), 0.212	1.1 (1.0, 1.1)*, 0.029	1.0 (0.9, 1.1),0.786
BMI (kg/m^2^)	1.1 (1.0, 1.1)*, 0.008	1.1 (1.0, 1.1)*, 0.046	1.1 (1.0, 1.1), 0.075
AMH (ng/ml)	1.0 (0.9, 1.1), 0.778	0.9 (0.8, 1.1), 0.282	1.0 (0.8, 1.1), 0.515
bFSH (mIU/ml)	0.9 (0.9, 1.0)*, 0.046	0.9 (0.9, 1.0), 0.275	1.0 (0.9, 1.1), 0.754
AFC	1.0 (1.0, 1.1), 0.066	1.0 (1.0, 1.0), 0.694	1.0 (1.0, 1.1), 0.994
Infertility factors (%)			
Tube	1.0	1.0	1.0
PCOS	1.4 (0.7, 3.0), 0.365	1.4 (0.5, 3.6), 0.525	1.3 (0.4, 4.6), 0.647
Endometriosis	1.0 (0.3, 2.8), 0.969	0.4 (0.1, 2.9), 0.365	0.0 (0.0, Inf), 0.986
POI	1.6 (0.6, 4.1), 0.324	2.1 (0.7, 6.2), 0.170	3.2 (0.9, 11.9), 0.077
Ovulation disorder	0.6 (0.1, 4.6), 0.642	0.9 (0.1, 7.0), 0.934	3.0 (0.3, 26.8), 0.319
Male	1.1 (0.7, 1.6), 0.703	1.1 (0.7, 1.9), 0.658	0.0 (0.0, Inf), 0.901
Unknown	0.0 (0.0, Inf), 0.972	0.0 (0.0, Inf), 0.978	1.0 (0.5, 1.9), 0.988
Diagnosis (%)			
Primary infertility	1.0	1.0	1.0
Secondary infertility	1.0 (0.7, 1.4), 0.888	1.5 (0.9, 2.3), 0.100	0.8 (0.5, 1.4), 0.477
Protocol(%)			
GnRH-a	1.0	1.0	1.0
GnRH-ant	0.9 (0.6, 1.4), 0.643	1.5 (0.8, 2.6), 0.188	1.4 (0.7, 3.0), 0.319
Long GnRH-a	0.8 (0.6, 1.2), 0.345	0.9 (0.5, 1.5), 0.697	0.9 (0.5, 1.7), 0.717
Average Gn dose (IU/day)	1.0 (1.0, 1.0), 0.683	1.0 (1.0, 1.0), 0.573	1.0 (1.0, 1.0), 0.537
No. of oocytes	1.0 (1.0, 1.0), 0.774	1.0 (0.9, 1.0), 0.432	1.0 (0.9, 1.1), 0.740
No. of 2 PN	1.0 (1.0, 1.1), 0.704	1.0 (0.9, 1.1), 0.952	1.0 (0.9, 1.1), 0.776
No. of 2 PN cleavage	1.0 (1.0, 1.1), 0.605	1.0 (0.9, 1.1), 0.928	1.0 (0.9, 1.1), 0.727
No. of available embryos	1.0 (0.9, 1.1), 0.462	1.0 (0.9, 1.2), 0.522	1.1 (0.9, 1.2), 0.327
Number of ET	1.6 (0.7, 3.6), 0.24	1.5 (0.5, 4.2), 0.427	1.0 (0.3, 3.5), 0.940
Progesterone (ng/ml)	0.9 (0.6, 1.3), 0.534	0.7 (0.4, 1.2), 0.256	1.0 (0.5, 1.9), 0.990

AMH, anti-Müllerian hormone; AFC, antral follicles; bFSH, basic follicle stimulating hormone; BMI, body mass index; CI, confidence interval; GnRH-a, gonadotropin-releasing hormone analogue; GnRH-ant, GnRH, antagonist; Inf, Infinity; OR, odds ratio; PCOS, polycystic ovary syndrome; POI, primary ovarian insufficiency; 2 PN, two pronuclear embryos; ET, embryo transfer. *p < 0.05.

**TABLE 6:** Risk factors for early abortion rate.

Risk factors	Early abortion rate	OR (95% CI), *p*	
	HCG0 (*n* = 2,404)	HCG-1 (*n* = 1,584)	HCG-2 (*n* = 800)
Age (years)	1.1 (1.0, 1.1)*, 0.012	1.1 (1.0, 1.2)*, 0.004	1.0 (1.0, 1.1), 0.369
BMI (kg/m^2^)	1.1 (1.0, 1.1)*, 0.033	1.1 (1.0, 1.1), 0.111	1.1 (1.0, 1.1), 0.165
AMH (ng/ml)	1.0 (0.9, 1.1), 0.642	0.9 (0.8, 1.0), 0.138	0.9 (0.8, 1.1), 0.266
bFSH (mIU/ml)	0.9 (0.8, 1.0), 0.096	1.0 (0.9, 1.1), 0.390	1.0 (0.9, 1.1), 0.823
AFC	1.0 (1.0, 1.1), 0.175	1.0 (1.0, 1.0), 0.810	1.0 (0.9, 1.0), 0.564
Infertility factors (%)			
Tube	1.0	1.0	1.0
PCOS	1.7 (0.8, 3.9), 0.184	1.8 (0.7, 4.8), 0.231	1.8 (0.5, 6.3), 0.364
Endometriosis	1.0 (0.3, 3.3), 0.971	0.5 (0.1, 3.9), 0.531	0.0 (0.0, Inf), 0.986
POI	2.3 (0.9, 5.9), 0.092	2.8 (0.9, 8.4), 0.062	4.3 (1.2, 16.2)*, 0.029
Ovulation disorder	0.9 (0.1, 6.6), 0.898	1.2 (0.2, 9.3), 0.849	4.1 (0.5, 36.2), 0.210
Male	1.1 (0.7, 1.7), 0.663	1.2 (0.7, 2.1), 0.572	0.0 (0.0, Inf), 0.789
Unknown	0.0 (0.0, Inf), 0.973	0.0 (0.0, Inf), 0.979	0.9 (0.4, 1.9), 0.989
Diagnosis (%)			
Primary infertility	1.0	1.0	1.0
Secondary infertility	0.9 (0.6, 1.3), 0.579	1.1 (0.7, 1.9), 0.605	0.8 (0.4, 1.5), 0.499
Protocol (%)			
GnRH-a	1.0	1.0	1.0
GnRH-ant	1.1 (0.7, 1.9), 0.687	1.7 (0.9, 3.1), 0.083	1.6 (0.7, 3.4), 0.263
Long GnRH-a	0.8 (0.5, 1.3), 0.461	0.7 (0.4, 1.3), 0.300	0.8 (0.4, 1.7), 0.545
Average Gn dose (IU/day)	1.0 (1.0, 1.0), 0.200	1.0 (1.0, 1.0), 0.080	1.0 (1.0, 1.0), 0.622
No. of oocytes	1.0 (1.0, 1.0), 0.950	1.0 (0.9, 1.0), 0.252	1.0 (0.9, 1.1), 0.728
No. of 2 PN	1.0 (1.0, 1.1), 0.684	1.0 (0.9, 1.1), 0.839	1.0 (0.9, 1.1), 0.928
No. of 2 PN cleavage	1.0 (1.0, 1.1), 0.591	1.0 (0.9, 1.1), 0.939	1.0 (0.9, 1.1), 0.983
No. of available embryos	1.0 (0.9, 1.1), 0.843	1.0 (0.9, 1.1), 0.761	1.0 (0.9, 1.2), 0.643
Number of ET	1.4 (0.6, 3.5), 0.420	1.2 (0.4, 3.3), 0.735	0.8 (0.2, 2.7), 0.703
Progesterone (ng/ml)	1.0 (0.6, 1.5), 0.992	0.8 (0.5, 1.5), 0.525	1.2 (0.6, 2.5), 0.600

AMH, anti-Müllerian hormone; AFC, antral follicles; bFSH, basic follicle stimulating hormone; BMI, body mass index; CI, confidence interval; GnRH-a, gonadotropin-releasing hormone analogue; GnRH-ant, GnRH, antagonist; OR, odds ratio; PCOS, polycystic ovary syndrome; POI, primary ovarian insufficiency; 2 PN, two pronuclear embryos; ET, embryo transfer. *p < 0.05.

The mean level of serum progesterone on HCG0 day was 1.1 ± 0.5 ng/ml. There were significant associations of the live birth rate with age, AMH, AFC, male factor, COS protocol, average Gn dosage, number of oocytes, number of 2 PN embryos, number of 2 PN cleavage embryos, number of available embryos, or number of transferred embryos (*p* < 0.05) (Table 4), whereas AFC, male factor, number of oocytes, number of 2 PN embryos, number of 2 PN cleavage embryos, number of available embryos, and number of transferred embryos were significantly associated with the clinical pregnancy rate (*p* < 0.05) (Table 2). Age, AFC, male factor, average Gn dosage, number of oocytes, number of 2 PN embryos, number of 2 PN cleavage embryos, number of available embryos, number of transferred embryos, and progesterone level were significantly (*p* < 0.05) correlated with the intrauterine pregnancy rate (Table 3). Only BMI and bFSH were significantly (*p* < 0.05) associated with the abortion rate, and age and BMI were significantly (*p* < 0.05) associated with the early abortion rate (Tables 5, 6).

The mean level of serum progesterone on HCG-1 day was 1.0 ± 0.4 ng/ml. Age, AMH, AFC, male factor, COS protocol, average Gn dose, number of oocytes, number of 2 PN embryos, number of 2 PN cleavage embryos, number of available embryos, and number of transferred embryos were significantly (*p* < 0.05) correlated with the live birth rate (Table 4), whereas AFC, PCOS, male factor, average Gn dose, number of oocytes, number of 2 PN embryos, number of 2 PN cleavage embryos, number of available embryos, number of transferred embryos, and progesterone level were significantly (*p* < 0.05) associated with the clinical pregnancy rate (Table 2). Age, AFC, male factor, average Gn dose, number of oocytes, number of 2 PN embryos, number of 2 PN cleavage embryos, number of available embryos, number of transferred embryos, and progesterone level were significantly (*p* < 0.05) correlated with the intrauterine pregnancy rate (Table 3). Age and BMI were significantly (*p* < 0.05) related to the abortion rate, and age was significantly (*p* < 0.05) related to the early abortion rate (Tables 5, 6).

The mean level of serum progesterone on HCG-2 day was 0.9 ± 0.4 ng/ml. Age, AFC, unknown infertility, and number of transferred embryos were significantly (*p* < 0.05) associated with the live birth rate (Table 4). AFC, unknown infertility, number of available embryos, number of transferred embryos, and progesterone level were significantly (*p* < 0.05) related to the clinical pregnancy rate (Table 2). Age, unknown infertility, number of available embryos, number of transferred embryos, and progesterone level were significantly (*p* < 0.05) related to the intrauterine pregnancy rate (Table 3). Premature ovarian insufficiency (POI) was significantly (*p* < 0.05) associated with the early abortion rate (Table 6). No risk factors were found to be significantly associated with abortion.

### Multivariate Logistic Regression Analysis

After adjustment of variables affecting the association between the progesterone level and pregnancy outcomes, there were 2,089 patients on HCG0, 1,399 on HCG-1, and 680 on HCG-2. In model I, the potential confounders were chosen based on the clinical experience: age, BMI, AMH, AFC, number of oocytes, and number of embryos transferred. In model II, the following variables were adjusted based on the clinical experience as well as results of the univariate analysis: age, BMI, AMH, AFC, factors of infertility, COS protocol, average Gn dosage, number of oocytes, number of 2 PN embryos, number of 2 PN cleavage embryos, number of available embryos, and number of transferred embryos. The progesterone level on HCG0 was an independent risk factor for clinical pregnancy rate (OR = 0.8, 95% CI: 0.6–0.9, *p* = 0.005), intrauterine pregnancy rate (OR = 0.7, 95% CI: 0.6–0.9, *p* = 0.002), and live birth rate (OR = 0.8, 95% CI: 0.6–1.0, *p* = 0.024). The progesterone level on HCG-1 was an independent risk factor for clinical pregnancy rate (OR = 0.7, 95% CI: 0.6–0.9, *p* = 0.010), intrauterine pregnancy rate (OR = −0.1, 95% CI: 0.2–0.0, *p* = 0.002), and live birth rate (OR = 0.7, 95% CI: 0.6–1.0, *p* = 0.026, model I). The progesterone level on HCG-2 was an independent risk factor for clinical pregnancy rate (OR = 0.7, 95% CI: 0.5–1.0, *p* = 0.032) and intrauterine pregnancy rate (OR = −0.1, 95% CI: 0.2–0.0, *p* = 0.026). The progesterone level in the late follicular phase had no significant (*p* > 0.05) predictive effect on the abortion rate and early abortion rate (Table 7).

**TABLE 7:** Multivariate logistic regression analysis after eliminating confounding factors.

	Clinical pregnancy rate		Intrauterine pregnancy rate		Live birth rate		Abortion rate		Early rate	Abortion
	OR (95%CI)	*p*	OR (95%CI)	*p*	OR (95%CI)	*p*	OR (95%CI)	*p*	OR (95%CI)	*p*
Non-adjusted										
HCG0 (*n* = 2,404)	0.9 (0.7, 1.0)	0.069	0.8 (0.7, 1.0)	0.037	0.9 (0.8, 1.1)	0.377	0.9 (0.6, 1.3)	0.534	1.0 (0.6, 1.5)	0.992
HCG-1 (*n* = 1,584)	0.8 (0.6, 1.0)	0.049	−0.0 (−0.1, −0.0)	0.018	0.9 (0.7, 1.1)	0.311	0.7 (0.4, 1.2)	0.256	0.8 (0.5, 1.5)	0.525
HCG-2 (*n* = 800)	0.7 (0.5, 1.0)	0.024	−0.1 (−0.2,−0.0)	0.025	0.7 (0.5, 1.0)	0.083	1.0 (0.5, 1.9)	0.990	1.2 (0.6, 2.5)	0.600
Adjusted I										
HCG0 (*n* = 2089)	0.8 (0.6, 0.9)	0.007	0.7 (0.6, 0.9)	0.002	0.8 (0.6, 1.0)	0.017	1.0 (0.7, 1.6)	0.823	1.3 (0.8, 2.2)	0.262
HCG-1 (n = 1,399)	0.7 (0.6, 0.9)	0.009	−0.1 (−0.2,−0.0)	0.002	0.7 (0.6, 1.0)	0.026	1.0 (0.5, 1.8)	0.924	1.3 (0.7, 2.5)	0.440
HCG-2 (*n* = 680)	0.6 (0.4, 0.9)	0.021	−0.1 (−0.2,−0.0)	0.016	0.7 (0.5, 1.0)	0.049	0.9 (0.4, 1.9)	0.832	1.1 (0.5, 2.6)	0.793
Adjusted II										
HCG0 (*n* = 2089)	0.8 (0.6, 0.9)	0.005	0.7 (0.6, 0.9)	0.002	0.8 (0.6, 1.0)	0.024	1.0 (0.7, 1.6)	0.864	1.3 (0.8, 2.1)	0.306
HCG-1 (*n* = 1,399)	0.7 (0.6, 0.9)	0.010	−0.1 (−0.2,−0.0)	0.002	0.8 (0.6, 1.0)	0.064	0.9 (0.5, 1.7)	0.784	1.2 (0.6, 2.2)	0.656
HCG-2 (*n* = 680)	0.7 (0.5, 1.0)	0.032	−0.1 (−0.2,−0.0)	0.026	0.7 (0.5, 1.0)	0.068	0.9 (0.4, 1.9)	0.829	1.1 (0.5, 2.5)	0.824

Adjusted I: age, BMI, AMH, AFC, number of oocytes, and number of ET were adjusted. Adjusted II: age, BMI, AMH, AFC, infertility factors, protocol, average Gn dose, number of oocytes, number of 2 PN, embryos, number of 2 PN, cleavage embryos, number of available embryos, and number of ET were adjusted; AMH, anti-Müllerian hormone; AFC, antral follicles; BMI, body mass index; OR, odds ratio; CI, confidence interval.

### Smooth Curve Fitting

**FIGURE 1:**
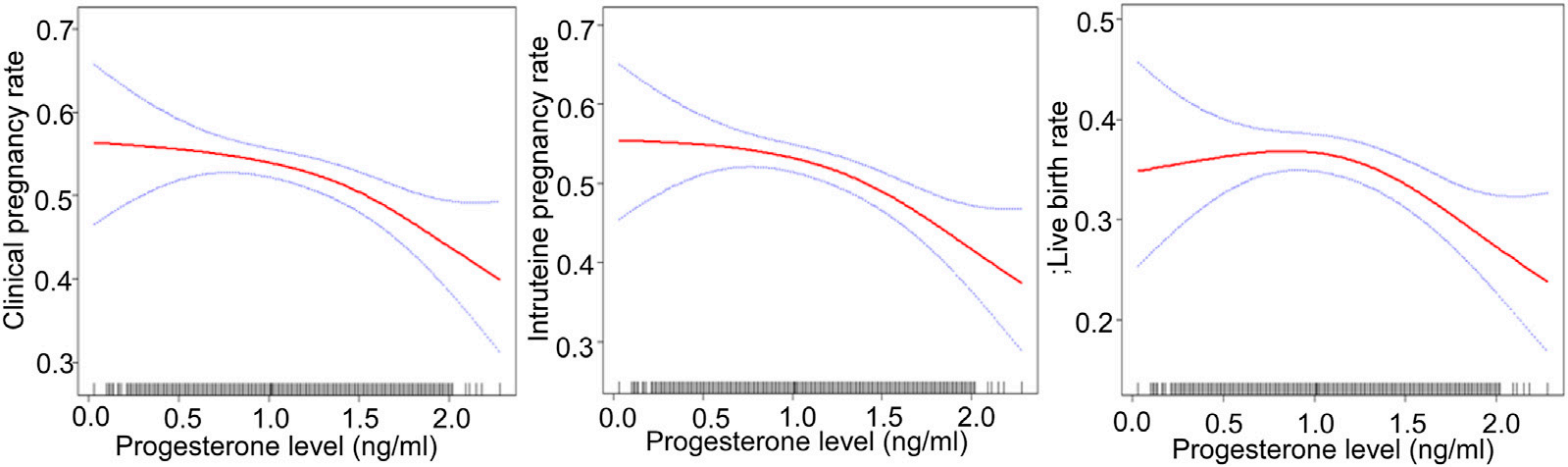
Association of progesterone levels on the day of HCG triggering with the IVF outcomes including the clinical pregnancy rate, intrauterine pregnancy rate, and live birth rate. Adjusted for age, BMI, AMH, AFC, infertility factors, protocol, average Gn dose, number of oocytes, number of 2 PN embryos, number of 2 PN cleavage embryos, number of available embryos, and number of ET. HCG, human chorionic gonadotropin; IVF, *in vitro* fertilization; BMI, body mass index, AFC, antral follicles; AMH, anti-Müllerian hormone; 2 PN, pronucleus; Gn, gonadotropin.

**FIGURE 2:**
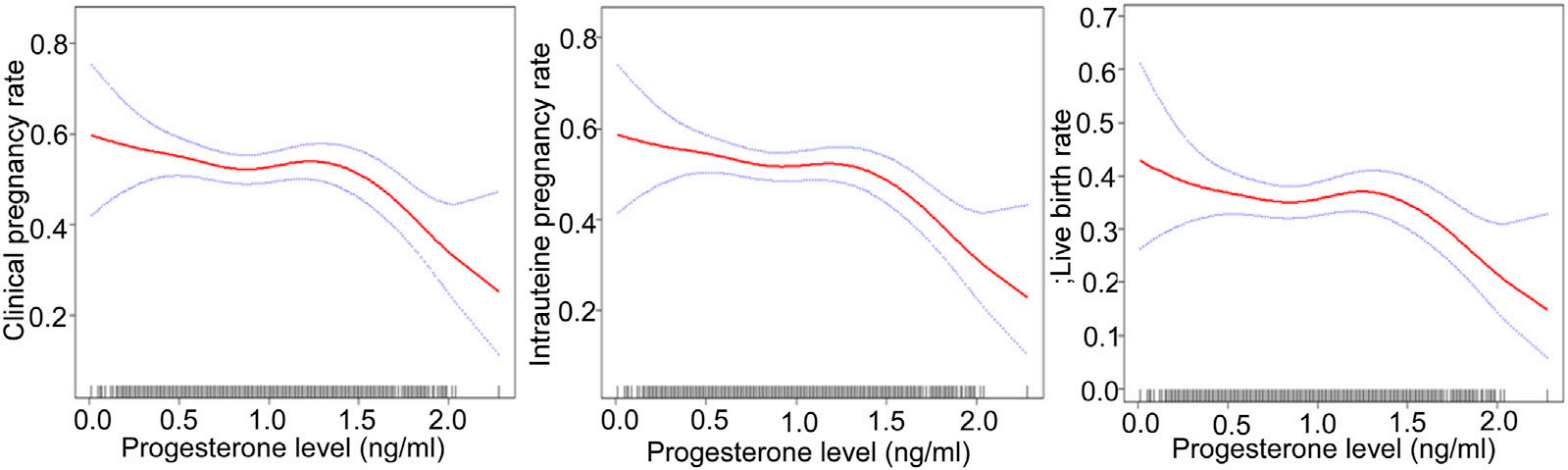
Association of progesterone levels on the day before HCG triggering with the IVF outcomes including the clinical pregnancy rate, the intrauterine pregnancy rate, and the live birth rate. The association of progesterone levels with the clinical and intrauterine pregnancy rate was calculated after adjustment for age, BMI, AMH, AFC, infertility factors, protocol, average Gn dose, number of oocytes, number of 2 PN embryos, number of 2 PN cleavage embryos, number of available embryos, and number of ET. The live birth rate was calculated after adjustment for age, BMI, AMH, AFC, number of oocytes, and number of ET. HCG, human chorionic gonadotropin; IVF, *in vitro* fertilization; BMI, body mass index, AFC, antral follicles; AMH, anti-Müllerian hormone; 2 PN, pronucleus; Gn, gonadotropin; ET, embryo transfer.

**FIGURE 3:**
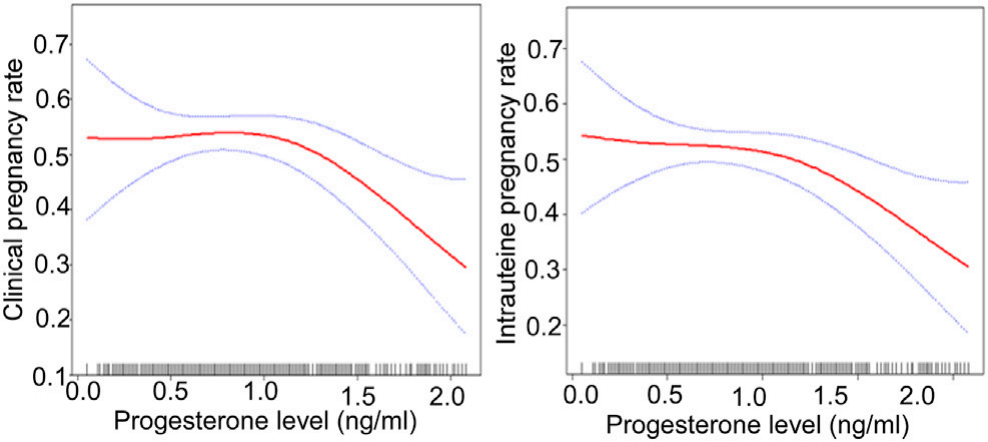
The association of the progesterone level on 2 days before HCG triggering with the IVF outcomes including the clinical pregnancy rate and the intrauterine pregnancy rate. Adjusted for age, BMI, AMH, AFC, infertility factors, protocol, average Gn dose, number of oocytes, number of 2 PN embryos, number of 2 PN cleavage embryos, number of available embryos, and number of ET. HCG, human chorionic gonadotropin; IVF, *in vitro* fertilization; BMI, body mass index, AFC, antral follicles; AMH, anti-Müllerian hormone; 2 PN, pronucleus; Gn, gonadotropin; ET, embryo transfer.

After adjusting for confounding factors, there was a non-linear relationship of the HCG0 or HCG-1 progesterone levels with the clinical pregnancy rate, intrauterine pregnancy rate, and live birth rate (Figures 1, 2). The HCG-2 progesterone level had a non-linear relationship with the clinical pregnancy rate and intrauterine pregnancy rate (Figure 3). When the progesterone level exceeded a certain level, the clinical pregnancy rate, intrauterine pregnancy rate, and live birth rate all showed a rapid downward trend.

### Threshold Effect Analysis

The threshold effect was analyzed after adjusting for confounding factors. When the progesterone level was ≥1.5 ng/ml on HCG0 day, with a 1 ng/ml increase in the progesterone level, the clinical pregnancy rate decreased by 60% (95% CI: 0.2–0.7, *p* = 0.004), the intrauterine pregnancy rate decreased by 70% (95% CI: 0.2–0.7, *p* = 0.003), and the live birth rate decreased by 70% (95% CI: 0.1–0.7, *p* = 0.004).

When the progesterone was ≥1.6 ng/ml on HCG-1 day, with a 1 ng/ml increase in the progesterone level, the clinical pregnancy rate decreased by 90% (95% CI: 0.0–0.5, *p* = 0.003), and the intrauterine pregnancy rate decreased by 90% (95% CI: 0.0–0.5, *p* = 0.001) when the progesterone level was ≥1.5 ng/ml. Similarly, the live birth rate decreased by 90% (95% CI: 0.0–0.6, *p* = 0.015) when the progesterone level was ≥1.7 ng/ml.

On HCG-2 day when the progesterone was ≥1.2 ng/ml, the clinical pregnancy rate decreased by 80% (95% CI: 0.1–0.6, *p* = 0.003), and the intrauterine pregnancy rate decreased by 70% (95% CI: 0.1–0.7, *p* = 0.007) with a 1 ng/ml increase in the progesterone level (Table 8).

**TABLE 8:** Threshold effect analysis of the progesterone level on IVF outcomes.

	OR	95% CI	*p*
Clinical pregnancy rate			
Pr ≥ 1.5(HCG0)	0.4	(0.2, 0.7)	0.004
Pr ≥ 1.6(HCG-1)	0.1	(0.0, 0.5)	0.003
Pr ≥ 1.2(HCG-2)	0.2	(0.1, 0.6)	0.003
Intrauterine pregnancy rate			
Pr ≥ 1.5(HCG0)	0.3	(0.2, 0.7)	0.003
Pr ≥ 1.5(HCG-1)	0.1	(0.0, 0.5)	0.001
Pr ≥ 1.2(HCG-2)	0.3	(0.1, 0.7)	0.007
Live birth rate			
Pr ≥ 1.5(HCG0)	0.3	(0.1, 0.7)	0.004
Pr ≥ 1.7(HCG-1)#	0.1	(0.0, 0.6)	0.015

#Adjusted for age, BMI, AMH, AFC, number of oocytes, and number of ET. pr, progesterone; HCG, human chorionic gonadotropin.

## Discussion

This study discovered that the increase of progesterone level during the late follicular phase in the COS process had a negative effect on the early pregnancy outcome and live birth rate in the IVF fresh cycle. However, currently, no consensus has been reached on the statistical methods for determining the critical value of serum progesterone elevation, and most researchers used the receiver operating characteristic (ROC) curves for analysis. Since the relationship between the serum progesterone level and the pregnancy outcome is not linear (Saleh et al., 2009; Bosch et al., 2010), the area under the ROC curve may not be the most suitable analysis method. Therefore, our study applied the smooth curve fitting and threshold effect analysis for the first time to determine the quantitative relationship between the increase in the progesterone level during the late follicular period and the pregnancy outcome of IVF fresh cycle.

To date, the largest meta-analysis of more than 60,000 IVF-ET cycles stratified according to different progesterone thresholds found that once the progesterone level exceeded 0.8 ng/ml, the increase in progesterone was significantly negatively correlated with pregnancy outcome (Venetis et al., 2013). When all the data were integrated, the threshold for increased progesterone that had the greatest impact on the pregnancy rate was between 1.5 ng/ml and 1.75 ng/ml (OR: 0.64, 95% CI: 0.54–0.76; *p* < 0.001) (Venetis et al., 2013). In addition, there did not seem to be a negative correlation between the progesterone elevation on the day of HCG triggering in fresh cycles and the pregnancy rate of frozen–thawed embryos transferred from this cycle, suggesting that elevated progesterone did not affect the quality of oocytes.

The study by Xiong et al. showed that the progesterone levels higher than 1.7 ng/ml on the day of HCG triggering affected the epigenetic modification of endometrium, including glandular epithelium, luminal epithelium, and stroma in the peri-implantation period, which may disrupt the endometrial receptivity and lead to a decrease in pregnancy rate (Xiong et al., 2017). However, some recent research has found a significant difference in the top embryo quality (TEQ) rate between serum progesterone levels <2.0 ng/ml and >2.0 ng/ml. Obviously, progesterone levels higher than 2.0 ng/ml before oocyte maturation were harmful to the quality of oocytes (Huang et al., 2016). Studies emphasized that elevated progesterone levels resulted in a potential decline in high-quality embryos and even a decrease in the cumulative pregnancy rates (Vanni et al., 2017; Racca et al., 2018). The latest research showed a significant negative correlation in the progesterone concentration exceeding 1 ng/ml with the proportion of high-quality embryos and the implantation rate (Simon et al., 2019).

In a study with 1,784 women, the clinical pregnancy rate decreased with the increase of high progesterone exposure time after dividing progesterone elevation (>1 ng/ml) time into three groups: 0, one to two, and ≥3 days (OR: 0.77, 95% CI: 0.66–0.89, *p* = 0.001). Therefore, not only the presence of high progesterone, but also the duration of high progesterone exposure seems to have a negative effect on the outcome of IVF (Huang et al., 2012). Our study found that the increase of serum progesterone concentration in different periods during the late follicular phase had adverse effects on clinical pregnancy outcome. The earlier the progesterone elevation, the lower the threshold and the greater the impact on pregnancy outcome. When the progesterone level was ≥1.5 ng/ml on HCG0 day, with a 1 ng/ml increase of the progesterone level, the clinical pregnancy rate decreased by 60% (95% CI: 0.2–0.7, *p* = 0.004), and the intrauterine pregnancy rate decreased by 70% (95% CI: 0.2–0.7, *p* = 0.003). When the progesterone level was ≥1.6 ng/ml on HCG-1 day, with a 1 ng/ml increase of progesterone, the clinical pregnancy rate decreased by 90% (95% CI: 0.0–0.5, *p* = 0.003). When the progesterone level was ≥1.5 ng/ml, the intrauterine pregnancy rate decreased by 90% (95% CI: 0.0–0.5, *p* = 0.001) with a 1 ng/ml increase in the progesterone level. When the progesterone level was ≥1.2 ng/ml on HCG-2 day, with a 1 ng/ml increase of progesterone, the clinical pregnancy rate decreased by 80% (95% CI: 0.1–0.6, *p* = 0.003), and the intrauterine pregnancy rate decreased by 70% (95% CI: 0.1–0.7, *p* = 0.007). However, our study did not find significant effects of elevated progesterone on abortion, including early abortion.

In the study by Santos-Ribeiro et al. focusing on exploring the relationship between the progesterone level on the day of HCG administration with GnRH antagonist protocol and the live birth rate, it was found that the lower (≤0.50 ng/ml) and upper (>1.5 ng/ml) limits of the progesterone level could significantly reduce the live birth rate (Santos-Ribeiro et al., 2014). One study that included 817 IVF fresh cycles with single birth showed that with the increase in the progesterone level, especially when the progesterone level was >2.0 ng/ml, the average birth weight decreased significantly (Ibrahim et al., 2017). A recent study suggested that in a cohort of women undergoing IVF, the serum concentration of progesterone in the range of 0.64–1.04 ng/ml (RR: 1.6, 95% CI: 1.1–2.5) on the day of HCG administration was significantly associated with an increased risk of ischemic placental disease (IPD), which might lead to fetal growth restriction; however, this was not seen in women with the highest progesterone levels (Modest et al., 2021). Our study found that regardless of the routine COS protocols, with a 1 ng/ml increase in the progesterone level on HCG0 day, the live birth rate decreased by 70% (95% CI: 0.1–0.7, *p* = 0.004) when the progesterone level was ≥1.5 ng/ml. When the progesterone level was ≥1.7 ng/ml on HCG-1 day, with a 1 ng/ml increase in the progesterone level, the live birth rate decreased by 90% (95% CI: 0.0–0.6, *p* = 0.015). However, the decrease in progesterone levels caused no significant adverse effects on the live birth rate. At present, the mechanism by which a high progesterone level leads to a decrease in the live birth rate and low birth weight is not clear, and the decrease of the progesterone level may have an impact on fetal development itself, or may be related to the internal factors of infertility and super-physiological hormone levels.

It has been shown that reducing the intensity of stimulation in the late follicular phase of COS could decrease the incidence of progesterone elevation (Lawrenz et al., 2016). The duration of COS should not be prolonged or exceeded beyond the optimal standard of final oocyte maturation. In this regard, the key to ovarian stimulation in IVF is an individualized therapy, which means to select the appropriate Gn dose according to the patient’s ovarian reserve and BMI, and to adjust the Gn dose according to hormone levels and follicular development during COS. In addition, compared with fresh embryo transfers, the application of “freeze-all” strategy could improve the perinatal outcome, including a lower incidence of preterm delivery and improved build for gestational age infants (Maheshwari et al., 2012; Shapiro et al., 2014), besides eliminating the adverse effect of elevated progesterone levels on endometrial receptivity (Fatemi and Garcia-Velasco, 2015).

Our study is the first known study to quantify the relationship between progesterone elevation in the late follicular phase and pregnancy outcomes in the IVF fresh cycle by smoothing curve fitting and threshold effect analysis. An observational retrospective study demonstrated that different progesterone assays had a limited reproducibility and results depending on the specific assay used and the range of progesterone level (Lawrenz et al., 2018). Consequently, the reliability of progesterone thresholds, suggested by a previously published meta-analysis on the topic (Venetis et al., 2013), has to be questioned because studies using different assays were included in that meta-analysis. Due to the differences in the performance and efficacy of progesterone detection methods (Patton et al., 2014) and inherent changes in secretion, it may be wise for each center to define its own progesterone threshold according to its own test results and clinical data.

Although strict inclusion and exclusion criteria had been adopted and many potential confounders had been adjusted as far as possible in this study, there were still some limitations, including the retrospective study design, single center study, and Chinese patients enrolled only, which may all affect the generalization of the outcomes of our study. Future studies will have to resolve these issues for better outcomes.

In summary, an increase of the serum progesterone level in the late follicular phase reduces the clinical pregnancy rate, intrauterine pregnancy rate, and live birth rate of IVF fresh cycles. The earlier the progesterone level rises, the lower the threshold and the greater impact on pregnancy outcomes. Therefore, we should not only pay attention to the progesterone level on the HCG triggering day, but also focus on whether the progesterone level increases in the late follicular phase to prevent possible adverse effect on pregnancy. The pregnancy outcomes of IVF can be improved by reducing the intensity of ovarian stimulation and freezing all embryos when the progesterone level is ≥1.5 ng/ml. Nonetheless, further studies are necessary to confirm this outcome.

## Data Availability

The raw data supporting the conclusion of this article will be made available by the authors, without undue reservation.
